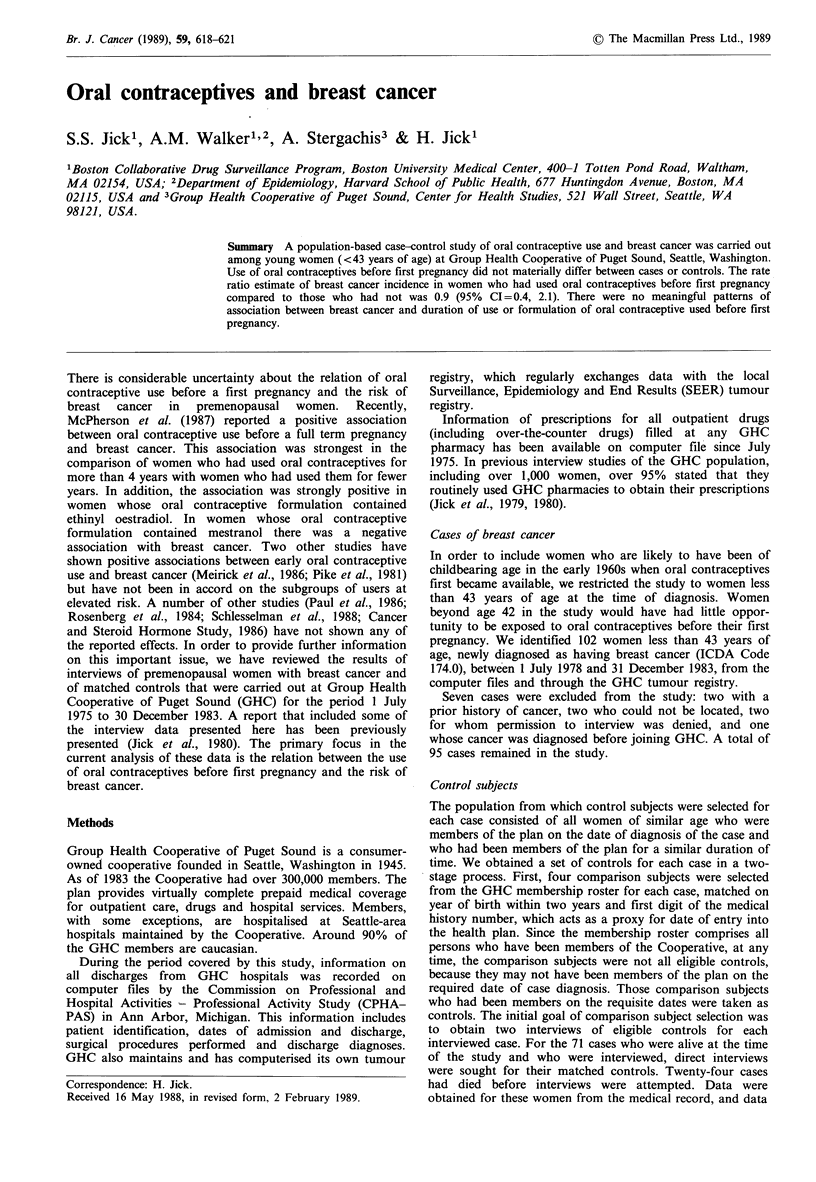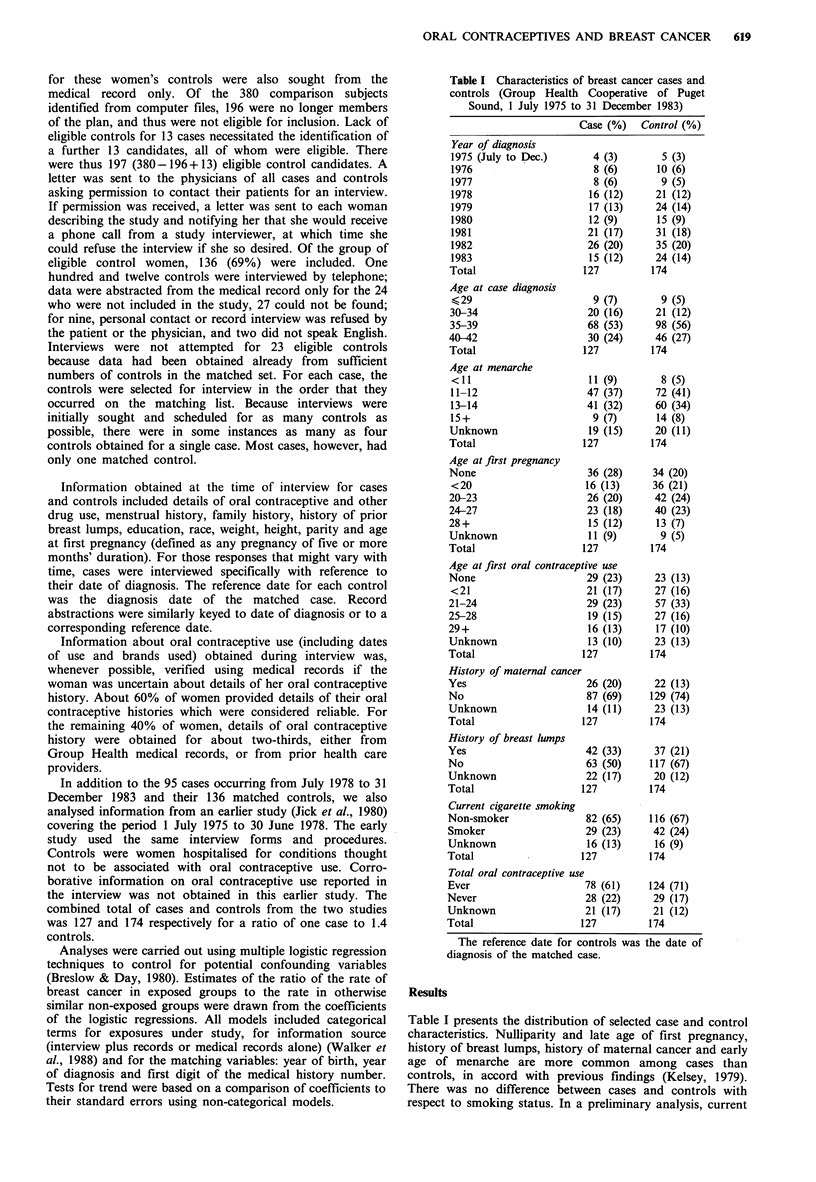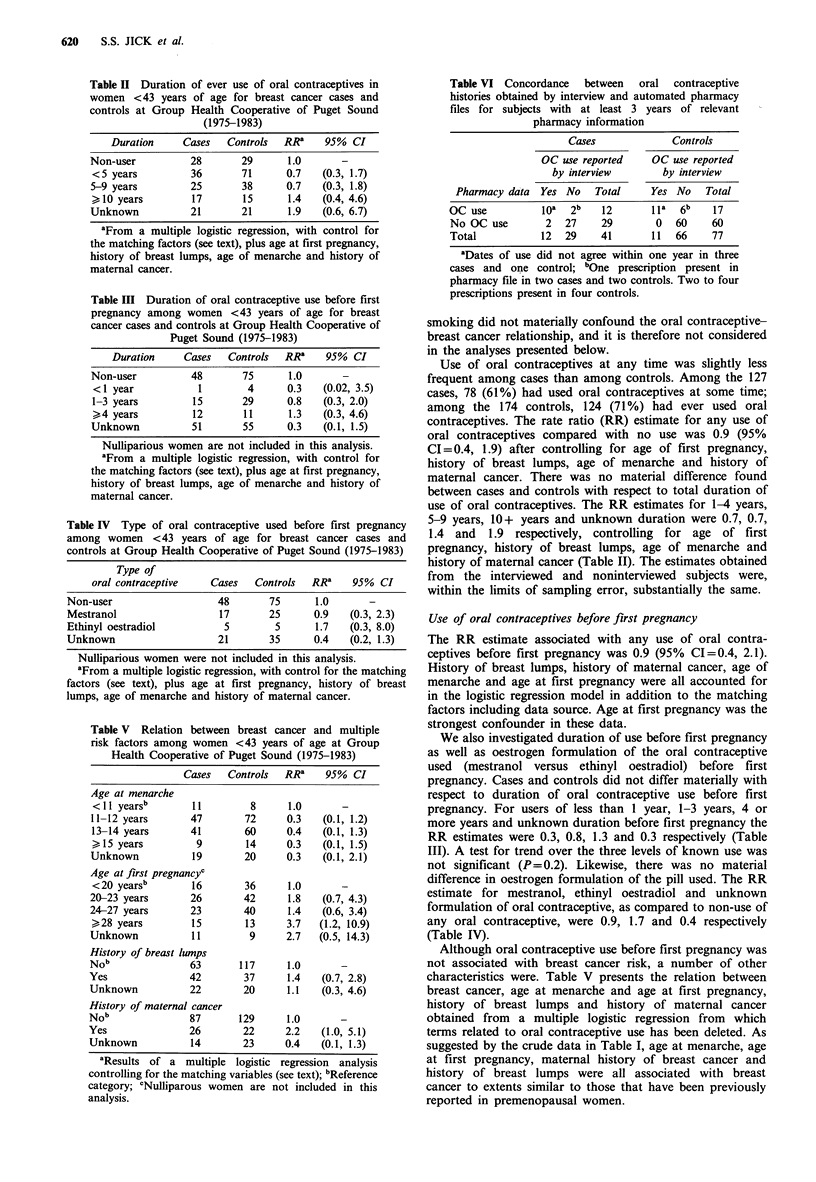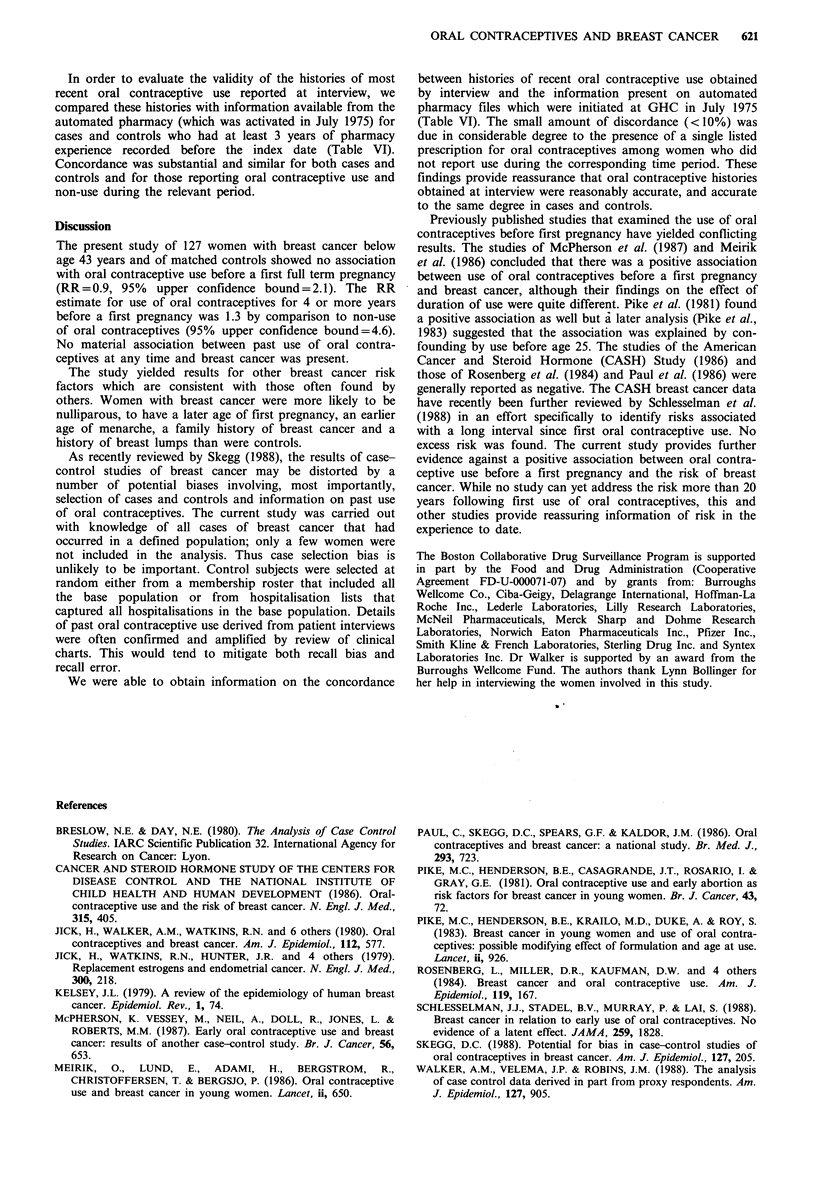# Oral contraceptives and breast cancer.

**DOI:** 10.1038/bjc.1989.125

**Published:** 1989-04

**Authors:** S. S. Jick, A. M. Walker, A. Stergachis, H. Jick

**Affiliations:** Boston Collaborative Drug Surveillance Program, Boston University Medical Center, Waltham, MA 02154.

## Abstract

A population-based case-control study of oral contraceptive use and breast cancer was carried out among young women (less than 43 years of age) at Group Health Cooperative of Puget Sound, Seattle, Washington. Use of oral contraceptives before first pregnancy did not materially differ between cases or controls. The rate ratio estimate of breast cancer incidence in women who had used oral contraceptives before first pregnancy compared to those who had not was 0.9 (95% CI = 0.4, 2.1). There were no meaningful patterns of association between breast cancer and duration of use or formulation of oral contraceptive used before first pregnancy.


Br. J. Cancer (1989), 59, 618–621 © The Macmillan Press Ltd., 1989

### Oral contraceptives and breast cancer

S.S. Jick[1], A.M. Walker[1,2], A. Stergachis[3] & H. Jick[1]

[1]*Boston Collaborative Drug Surveillance Program, Boston University Medical Center, 400–1 Totten Pond Road, Waltham, MA 02154, USA;* [2]*Department of Epidemiology, Harvard School of Public Health, 677 Huntingdon Avenue, Boston, MA 02115, USA and* [3]*Group Health Cooperative of Puget Sound, Center for Health Studies, 521 Wall Street, Seattle, WA 98121, USA.*

**Summary** A population-based case–control study of oral contraceptive use and breast cancer was carried out among young women (<43 years of age) at Group Health Cooperative of Puget Sound, Seattle, Washington. Use of oral contraceptives before first pregnancy did not materially differ between cases or controls. The rate ratio estimate of breast cancer incidence in women who had used oral contraceptives before first pregnancy compared to those who had not was 0.9 (95% CI = 0.4, 2.1). There were no meaningful patterns of association between breast cancer and duration of use or formulation of oral contraceptive used before first pregnancy.

There is considerable uncertainty about the relation of oral contraceptive use before a first pregnancy and the risk of breast cancer in premenopausal women. Recently, McPherson *et al.* (1987) reported a positive association between oral contraceptive use before a full term pregnancy and breast cancer. This association was strongest in the comparison of women who had used oral contraceptives for more than 4 years with women who had used them for fewer years. In addition, the association was strongly positive in women whose oral contraceptive formulation contained ethinyl oestradiol. In women whose oral contraceptive formulation contained mestranol there was a negative association with breast cancer. Two other studies have shown positive associations between early oral contraceptive use and breast cancer (Meirick *et al.*, 1986; Pike *et al.*, 1981) but have not been in accord on the subgroups of users at elevated risk. A number of other studies (Paul *et al.*, 1986; Rosenberg *et al.*, 1984; Schlesselman *et al.*, 1988; Cancer and Steroid Hormone Study, 1986) have not shown any of the reported effects. In order to provide further information on this important issue, we have reviewed the results of interviews of premenopausal women with breast cancer and of matched controls that were carried out at Group Health Cooperative of Puget Sound (GHC) for the period 1 July 1975 to 30 December 1983. A report that included some of the interview data presented here has been previously presented (Jick *et al.*, 1980). The primary focus in the current analysis of these data is the relation between the use of oral contraceptives before first pregnancy and the risk of breast cancer.

### Methods

Group Health Cooperative of Puget Sound is a consumer-owned cooperative founded in Seattle, Washington in 1945. As of 1983 the Cooperative had over 300,000 members. The plan provides virtually complete prepaid medical coverage for outpatient care, drugs and hospital services. Members, with some exceptions, are hospitalised at Seattle-area hospitals maintained by the Cooperative. Around 90% of the GHC members are caucasian.

During the period covered by this study, information on all discharges from GHC hospitals was recorded on computer files by the Commission on Professional and Hospital Activities – Professional Activity Study (CPHA-PAS) in Ann Arbor, Michigan. This information includes patient identification, dates of admission and discharge, surgical procedures performed and discharge diagnoses. GHC also maintains and has computerised its own tumour registry, which regularly exchanges data with the local Surveillance, Epidemiology and End Results (SEER) tumour registry.

Information of prescriptions for all outpatient drugs (including over-the-counter drugs) filled at any GHC pharmacy has been available on computer file since July 1975. In previous interview studies of the GHC population, including over 1,000 women, over 95% stated that they routinely used GHC pharmacies to obtain their prescriptions (Jick *et al.*, 1979, 1980).

### *Cases of breast cancer*

In order to include women who are likely to have been of childbearing age in the early 1960s when oral contraceptives first became available, we restricted the study to women less than 43 years of age at the time of diagnosis. Women beyond age 42 in the study would have had little opportunity to be exposed to oral contraceptives before their first pregnancy. We identified 102 women less than 43 years of age, newly diagnosed as having breast cancer (ICDA Code 174.0), between 1 July 1978 and 31 December 1983, from the computer files and through the GHC tumour registry.

Seven cases were excluded from the study: two with a prior history of cancer, two who could not be located, two for whom permission to interview was denied, and one whose cancer was diagnosed before joining GHC. A total of 95 cases remained in the study.

### *Control subjects*

The population from which control subjects were selected for each case consisted of all women of similar age who were members of the plan on the date of diagnosis of the case and who had been members of the plan for a similar duration of time. We obtained a set of controls for each case in a two-stage process. First, four comparison subjects were selected from the GHC membership roster for each case, matched on year of birth within two years and first digit of the medical history number, which acts as a proxy for date of entry into the health plan. Since the membership roster comprises all persons who have been members of the Cooperative, at any time, the comparison subjects were not all eligible controls, because they may not have been members of the plan on the required date of case diagnosis. Those comparison subjects who had been members on the requisite dates were taken as controls. The initial goal of comparison subject selection was to obtain two interviews of eligible controls for each interviewed case. For the 71 cases who were alive at the time of the study and who were interviewed, direct interviews were sought for their matched controls. Twenty-four cases had died before interviews were attempted. Data were obtained for these women from the medical record, and data

Correspondence: H. Jick.
Received 16 May 1988, in revised form, 2 February 1989.

for these women's controls were also sought from the medical record only. Of the 380 comparison subjects identified from computer files, 196 were no longer members of the plan, and thus were not eligible for inclusion. Lack of eligible controls for 13 cases necessitated the identification of a further 13 candidates, all of whom were eligible. There were thus 197 (380 − 196 + 13) eligible control candidates. A letter was sent to the physicians of all cases and controls asking permission to contact their patients for an interview. If permission was received, a letter was sent to each woman describing the study and notifying her that she would receive a phone call from a study interviewer, at which time she could refuse the interview if she so desired. Of the group of eligible control women, 136 (69%) were included. One hundred and twelve controls were interviewed by telephone; data were abstracted from the medical record only for the 24 who were not included in the study, 27 could not be found; for nine, personal contact or record interview was refused by the patient or the physician, and two did not speak English. Interviews were not attempted for 23 eligible controls because data had been obtained already from sufficient numbers of controls in the matched set. For each case, the controls were selected for interview in the order that they occurred on the matching list. Because interviews were initially sought and scheduled for as many controls as possible, there were in some instances as many as four controls obtained for a single case. Most cases, however, had only one matched control.

Information obtained at the time of interview for cases and controls included details of oral contraceptive and other drug use, menstrual history, family history, history of prior breast lumps, education, race, weight, height, parity and age at first pregnancy (defined as any pregnancy of five or more months' duration). For those responses that might vary with time, cases were interviewed specifically with reference to their date of diagnosis. The reference date for each control was the diagnosis date of the matched case. Record abstractions were similarly keyed to date of diagnosis or to a corresponding reference date.

Information about oral contraceptive use (including dates of use and brands used) obtained during interview was, whenever possible, verified using medical records if the woman was uncertain about details of her oral contraceptive history. About 60% of women provided details of their oral contraceptive histories which were considered reliable. For the remaining 40% of women, details of oral contraceptive history were obtained for about two-thirds, either from Group Health medical records, or from prior health care providers.

In addition to the 95 cases occurring from July 1978 to 31 December 1983 and their 136 matched controls, we also analysed information from an earlier study (Jick *et al.*, 1980) covering the period 1 July 1975 to 30 June 1978. The early study used the same interview forms and procedures. Controls were women hospitalised for conditions thought not to be associated with oral contraceptive use. Corroborative information on oral contraceptive use reported in the interview was not obtained in this earlier study. The combined total of cases and controls from the two studies was 127 and 174 respectively for a ratio of one case to 1.4 controls.

Analyses were carried out using multiple logistic regression techniques to control for potential confounding variables (Breslow & Day, 1980). Estimates of the ratio of the rate of breast cancer in exposed groups to the rate in otherwise similar non-exposed groups were drawn from the coefficients of the logistic regressions. All models included categorical terms for exposures under study, for information source (interview plus records or medical records alone) (Walker *et al.*, 1988) and for the matching variables: year of birth, year of diagnosis and first digit of the medical history number. Tests for trend were based on a comparison of coefficients to their standard errors using non-categorical models.

**Table I** Characteristics of breast cancer cases and controls (Group Health Cooperative of Puget Sound, 1 July 1975 to 31 December 1983)

| | Case (%) | *Control* (%) |
|---|---|---|
| *Year of diagnosis* | | |
| 1975 (July to Dec.) | 4 (3) | 5 (3) |
| 1976 | 8 (6) | 10 (6) |
| 1977 | 8 (6) | 9 (5) |
| 1978 | 16 (12) | 21 (12) |
| 1979 | 17 (13) | 24 (14) |
| 1980 | 12 (9) | 15 (9) |
| 1981 | 21 (17) | 31 (18) |
| 1982 | 26 (20) | 35 (20) |
| 1983 | 15 (12) | 24 (14) |
| Total | 127 | 174 |
| *Age at case diagnosis* | | |
| ≤29 | 9 (7) | 9 (5) |
| 30–34 | 20 (16) | 21 (12) |
| 35–39 | 68 (53) | 98 (56) |
| 40–42 | 30 (24) | 46 (27) |
| Total | 127 | 174 |
| *Age at menarche* | | |
| <11 | 11 (9) | 8 (5) |
| 11–12 | 47 (37) | 72 (41) |
| 13–14 | 41 (32) | 60 (34) |
| 15+ | 9 (7) | 14 (8) |
| Unknown | 19 (15) | 20 (11) |
| Total | 127 | 174 |
| *Age at first pregnancy* | | |
| None | 36 (28) | 34 (20) |
| <20 | 16 (13) | 36 (21) |
| 20–23 | 26 (20) | 42 (24) |
| 24–27 | 23 (18) | 40 (23) |
| 28+ | 15 (12) | 13 (7) |
| Unknown | 11 (9) | 9 (5) |
| Total | 127 | 174 |
| *Age at first oral contraceptive use* | | |
| None | 29 (23) | 23 (13) |
| <21 | 21 (17) | 27 (16) |
| 21–24 | 29 (23) | 57 (33) |
| 25–28 | 19 (15) | 27 (16) |
| 29+ | 16 (13) | 17 (10) |
| Unknown | 13 (10) | 23 (13) |
| Total | 127 | 174 |
| *History of maternal cancer* | | |
| Yes | 26 (20) | 22 (13) |
| No | 87 (69) | 129 (74) |
| Unknown | 14 (11) | 23 (13) |
| Total | 127 | 174 |
| *History of breast lumps* | | |
| Yes | 42 (33) | 37 (21) |
| No | 63 (50) | 117 (67) |
| Unknown | 22 (17) | 20 (12) |
| Total | 127 | 174 |
| *Current cigarette smoking* | | |
| Non-smoker | 82 (65) | 116 (67) |
| Smoker | 29 (23) | 42 (24) |
| Unknown | 16 (13) | 16 (9) |
| Total | 127 | 174 |
| *Total oral contraceptive use* | | |
| Ever | 78 (61) | 124 (71) |
| Never | 28 (22) | 29 (17) |
| Unknown | 21 (17) | 21 (12) |
| Total | 127 | 174 |

The reference date for controls was the date of diagnosis of the matched case.

### Results

Table I presents the distribution of selected case and control characteristics. Nulliparity and late age of first pregnancy, history of breast lumps, history of maternal cancer and early age of menarche are more common among cases than controls, in accord with previous findings (Kelsey, 1979). There was no difference between cases and controls with respect to smoking status. In a preliminary analysis, current

**Table II** Duration of ever use of oral contraceptives in women <43 years of age for breast cancer cases and controls at Group Health Cooperative of Puget Sound (1975–1983)

| *Duration* | *Cases* | *Controls* | *RR*[a] | *95% CI* |
|---|---|---|---|---|
| Non-user | 28 | 29 | 1.0 | – |
| <5 years | 36 | 71 | 0.7 | (0.3, 1.7) |
| 5–9 years | 25 | 38 | 0.7 | (0.3, 1.8) |
| ≥10 years | 17 | 15 | 1.4 | (0.4, 4.6) |
| Unknown | 21 | 21 | 1.9 | (0.6, 6.7) |

[a]From a multiple logistic regression, with control for the matching factors (see text), plus age at first pregnancy, history of breast lumps, age of menarche and history of maternal cancer.

**Table III** Duration of oral contraceptive use before first pregnancy among women <43 years of age for breast cancer cases and controls at Group Health Cooperative of Puget Sound (1975–1983)

| *Duration* | *Cases* | *Controls* | *RR*[a] | *95% CI* |
|---|---|---|---|---|
| Non-user | 48 | 75 | 1.0 | – |
| <1 year | 1 | 4 | 0.3 | (0.02, 3.5) |
| 1–3 years | 15 | 29 | 0.8 | (0.3, 2.0) |
| ≥4 years | 12 | 11 | 1.3 | (0.3, 4.6) |
| Unknown | 51 | 55 | 0.3 | (0.1, 1.5) |

Nulliparious women are not included in this analysis.
[a]From a multiple logistic regression, with control for the matching factors (see text), plus age at first pregnancy, history of breast lumps, age of menarche and history of maternal cancer.

**Table IV** Type of oral contraceptive used before first pregnancy among women <43 years of age for breast cancer cases and controls at Group Health Cooperative of Puget Sound (1975–1983)

| *Type of oral contraceptive* | *Cases* | *Controls* | *RR*[a] | *95% CI* |
|---|---|---|---|---|
| Non-user | 48 | 75 | 1.0 | – |
| Mestranol | 17 | 25 | 0.9 | (0.3, 2.3) |
| Ethinyl oestradiol | 5 | 5 | 1.7 | (0.3, 8.0) |
| Unknown | 21 | 35 | 0.4 | (0.2, 1.3) |

Nulliparious women were not included in this analysis.
[a]From a multiple logistic regression, with control for the matching factors (see text), plus age at first pregnancy, history of breast lumps, age of menarche and history of maternal cancer.

**Table V** Relation between breast cancer and multiple risk factors among women <43 years of age at Group Health Cooperative of Puget Sound (1975–1983)

| | *Cases* | *Controls* | *RR*[a] | *95% CI* |
|---|---|---|---|---|
| *Age at menarche* | | | | |
| <11 years[b] | 11 | 8 | 1.0 | – |
| 11–12 years | 47 | 72 | 0.3 | (0.1, 1.2) |
| 13–14 years | 41 | 60 | 0.4 | (0.1, 1.3) |
| ≥15 years | 9 | 14 | 0.3 | (0.1, 1.5) |
| Unknown | 19 | 20 | 0.3 | (0.1, 2.1) |
| *Age at first pregnancy*[c] | | | | |
| <20 years[b] | 16 | 36 | 1.0 | – |
| 20–23 years | 26 | 42 | 1.8 | (0.7, 4.3) |
| 24–27 years | 23 | 40 | 1.4 | (0.6, 3.4) |
| ≥28 years | 15 | 13 | 3.7 | (1.2, 10.9) |
| Unknown | 11 | 9 | 2.7 | (0.5, 14.3) |
| *History of breast lumps* | | | | |
| No[b] | 63 | 117 | 1.0 | – |
| Yes | 42 | 37 | 1.4 | (0.7, 2.8) |
| Unknown | 22 | 20 | 1.1 | (0.3, 4.6) |
| *History of maternal cancer* | | | | |
| No[b] | 87 | 129 | 1.0 | – |
| Yes | 26 | 22 | 2.2 | (1.0, 5.1) |
| Unknown | 14 | 23 | 0.4 | (0.1, 1.3) |

[a]Results of a multiple logistic regression analysis controlling for the matching variables (see text); [b]Reference category; [c]Nulliparous women are not included in this analysis.

**Table VI** Concordance between oral contraceptive histories obtained by interview and automated pharmacy files for subjects with at least 3 years of relevant pharmacy information

| | *Cases* | | | *Controls* | | |
|---|---|---|---|---|---|---|
| | *OC use reported by interview* | | | *OC use reported by interview* | | |
| *Pharmacy data* | *Yes* | *No* | *Total* | *Yes* | *No* | *Total* |
| OC use | 10[a] | 2[b] | 12 | 11[a] | 6[b] | 17 |
| No OC use | 2 | 27 | 29 | 0 | 60 | 60 |
| Total | 12 | 29 | 41 | 11 | 66 | 77 |

[a]Dates of use did not agree within one year in three cases and one control; [b]One prescription present in pharmacy file in two cases and two controls. Two to four prescriptions present in four controls.

smoking did not materially confound the oral contraceptive–breast cancer relationship, and it is therefore not considered in the analyses presented below.

Use of oral contraceptives at any time was slightly less frequent among cases than among controls. Among the 127 cases, 78 (61%) had used oral contraceptives at some time; among the 174 controls, 124 (71%) had ever used oral contraceptives. The rate ratio (RR) estimate for any use of oral contraceptives compared with no use was 0.9 (95% CI=0.4, 1.9) after controlling for age of first pregnancy, history of breast lumps, age of menarche and history of maternal cancer. There was no material difference found between cases and controls with respect to total duration of use of oral contraceptives. The RR estimates for 1–4 years, 5–9 years, 10+ years and unknown duration were 0.7, 0.7, 1.4 and 1.9 respectively, controlling for age of first pregnancy, history of breast lumps, age of menarche and history of maternal cancer (Table II). The estimates obtained from the interviewed and noninterviewed subjects were, within the limits of sampling error, substantially the same.

### *Use of oral contraceptives before first pregnancy*

The RR estimate associated with any use of oral contraceptives before first pregnancy was 0.9 (95% CI=0.4, 2.1). History of breast lumps, history of maternal cancer, age of menarche and age at first pregnancy were all accounted for in the logistic regression model in addition to the matching factors including data source. Age at first pregnancy was the strongest confounder in these data.

We also investigated duration of use before first pregnancy as well as oestrogen formulation of the oral contraceptive used (mestranol versus ethinyl oestradiol) before first pregnancy. Cases and controls did not differ materially with respect to duration of oral contraceptive use before first pregnancy. For users of less than 1 year, 1–3 years, 4 or more years and unknown duration before first pregnancy the RR estimates were 0.3, 0.8, 1.3 and 0.3 respectively (Table III). A test for trend over the three levels of known use was not significant ($P=0.2$). Likewise, there was no material difference in oestrogen formulation of the pill used. The RR estimate for mestranol, ethinyl oestradiol and unknown formulation of oral contraceptive, as compared to non-use of any oral contraceptive, were 0.9, 1.7 and 0.4 respectively (Table IV).

Although oral contraceptive use before first pregnancy was not associated with breast cancer risk, a number of other characteristics were. Table V presents the relation between breast cancer, age at menarche and age at first pregnancy, history of breast lumps and history of maternal cancer obtained from a multiple logistic regression from which terms related to oral contraceptive use has been deleted. As suggested by the crude data in Table I, age at menarche, age at first pregnancy, maternal history of breast cancer and history of breast lumps were all associated with breast cancer to extents similar to those that have been previously reported in premenopausal women.

In order to evaluate the validity of the histories of most recent oral contraceptive use reported at interview, we compared these histories with information available from the automated pharmacy (which was activated in July 1975) for cases and controls who had at least 3 years of pharmacy experience recorded before the index date (Table VI). Concordance was substantial and similar for both cases and controls and for those reporting oral contraceptive use and non-use during the relevant period.

### Discussion

The present study of 127 women with breast cancer below age 43 years and of matched controls showed no association with oral contraceptive use before a first full term pregnancy (RR = 0.9, 95% upper confidence bound = 2.1). The RR estimate for use of oral contraceptives for 4 or more years before a first pregnancy was 1.3 by comparison to non-use of oral contraceptives (95% upper confidence bound = 4.6). No material association between past use of oral contraceptives at any time and breast cancer was present.

The study yielded results for other breast cancer risk factors which are consistent with those often found by others. Women with breast cancer were more likely to be nulliparous, to have a later age of first pregnancy, an earlier age of menarche, a family history of breast cancer and a history of breast lumps than were controls.

As recently reviewed by Skegg (1988), the results of case–control studies of breast cancer may be distorted by a number of potential biases involving, most importantly, selection of cases and controls and information on past use of oral contraceptives. The current study was carried out with knowledge of all cases of breast cancer that had occurred in a defined population; only a few women were not included in the analysis. Thus case selection bias is unlikely to be important. Control subjects were selected at random either from a membership roster that included all the base population or from hospitalisation lists that captured all hospitalisations in the base population. Details of past oral contraceptive use derived from patient interviews were often confirmed and amplified by review of clinical charts. This would tend to mitigate both recall bias and recall error.

We were able to obtain information on the concordance between histories of recent oral contraceptive use obtained by interview and the information present on automated pharmacy files which were initiated at GHC in July 1975 (Table VI). The small amount of discordance (< 10%) was due in considerable degree to the presence of a single listed prescription for oral contraceptives among women who did not report use during the corresponding time period. These findings provide reassurance that oral contraceptive histories obtained at interview were reasonably accurate, and accurate to the same degree in cases and controls.

Previously published studies that examined the use of oral contraceptives before first pregnancy have yielded conflicting results. The studies of McPherson *et al.* (1987) and Meirik *et al.* (1986) concluded that there was a positive association between use of oral contraceptives before a first pregnancy and breast cancer, although their findings on the effect of duration of use were quite different. Pike *et al.* (1981) found a positive association as well but a later analysis (Pike *et al.*, 1983) suggested that the association was explained by confounding by use before age 25. The studies of the American Cancer and Steroid Hormone (CASH) Study (1986) and those of Rosenberg *et al.* (1984) and Paul *et al.* (1986) were generally reported as negative. The CASH breast cancer data have recently been further reviewed by Schlesselman *et al.* (1988) in an effort specifically to identify risks associated with a long interval since first oral contraceptive use. No excess risk was found. The current study provides further evidence against a positive association between oral contraceptive use before a first pregnancy and the risk of breast cancer. While no study can yet address the risk more than 20 years following first use of oral contraceptives, this and other studies provide reassuring information of risk in the experience to date.

The Boston Collaborative Drug Surveillance Program is supported in part by the Food and Drug Administration (Cooperative Agreement FD-U-000071-07) and by grants from: Burroughs Wellcome Co., Ciba-Geigy, Delagrange International, Hoffman-La Roche Inc., Lederle Laboratories, Lilly Research Laboratories, McNeil Pharmaceuticals, Merck Sharp and Dohme Research Laboratories, Norwich Eaton Pharmaceuticals Inc., Pfizer Inc., Smith Kline & French Laboratories, Sterling Drug Inc. and Syntex Laboratories Inc. Dr Walker is supported by an award from the Burroughs Wellcome Fund. The authors thank Lynn Bollinger for her help in interviewing the women involved in this study.

### References

BRESLOW, N.E. & DAY, N.E. (1980). *The Analysis of Case Control Studies.* IARC Scientific Publication 32. International Agency for Research on Cancer: Lyon.

CANCER AND STEROID HORMONE STUDY OF THE CENTERS FOR DISEASE CONTROL AND THE NATIONAL INSTITUTE OF CHILD HEALTH AND HUMAN DEVELOPMENT (1986). Oral-contraceptive use and the risk of breast cancer. *N. Engl. J. Med.*, **315**, 405.

JICK, H., WALKER, A.M., WATKINS, R.N. and 6 others (1980). Oral contraceptives and breast cancer. *Am. J. Epidemiol.*, **112**, 577.

JICK, H., WATKINS, R.N., HUNTER, J.R. and 4 others (1979). Replacement estrogens and endometrial cancer. *N. Engl. J. Med.*, **300**, 218.

KELSEY, J.L. (1979). A review of the epidemiology of human breast cancer. *Epidemiol. Rev.*, **1**, 74.

McPHERSON, K. VESSEY, M., NEIL, A., DOLL, R., JONES, L. & ROBERTS, M.M. (1987). Early oral contraceptive use and breast cancer: results of another case–control study. *Br. J. Cancer*, **56**, 653.

MEIRIK, O., LUND, E., ADAMI, H., BERGSTROM, R., CHRISTOFFERSEN, T. & BERGSJO, P. (1986). Oral contraceptive use and breast cancer in young women. *Lancet*, **ii**, 650.

PAUL, C., SKEGG, D.C., SPEARS, G.F. & KALDOR, J.M. (1986). Oral contraceptives and breast cancer: a national study. *Br. Med. J.*, **293**, 723.

PIKE, M.C., HENDERSON, B.E., CASAGRANDE, J.T., ROSARIO, I. & GRAY, G.E. (1981). Oral contraceptive use and early abortion as risk factors for breast cancer in young women. *Br. J. Cancer*, **43**, 72.

PIKE, M.C., HENDERSON, B.E., KRAILO, M.D., DUKE, A. & ROY, S. (1983). Breast cancer in young women and use of oral contraceptives: possible modifying effect of formulation and age at use. *Lancet*, **ii**, 926.

ROSENBERG, L., MILLER, D.R., KAUFMAN, D.W. and 4 others (1984). Breast cancer and oral contraceptive use. *Am. J. Epidemiol.*, **119**, 167.

SCHLESSELMAN, J.J., STADEL, B.V., MURRAY, P. & LAI, S. (1988). Breast cancer in relation to early use of oral contraceptives. No evidence of a latent effect. *JAMA*, **259**, 1828.

SKEGG, D.C. (1988). Potential for bias in case–control studies of oral contraceptives in breast cancer. *Am. J. Epidemiol.*, **127**, 205.

WALKER, A.M., VELEMA, J.P. & ROBINS, J.M. (1988). The analysis of case control data derived in part from proxy respondents. *Am. J. Epidemiol.*, **127**, 905.